# A Complex Case: Tubercular Pleural Effusion, Infarct, and Multisystem Involvement

**DOI:** 10.7759/cureus.68539

**Published:** 2024-09-03

**Authors:** Milly M Tadigiri, Arub Imam, Rishab J Martins

**Affiliations:** 1 Department of Surgery, Bharati Vidyapeeth Hospital, Pune, IND; 2 Department of Medicine, Bharati Vidyapeeth Hospital, Pune, IND

**Keywords:** complexity, infarct, tuberculosis, pleural effusion, comorbidities

## Abstract

Tuberculous pleural effusion, the second most common type of tuberculosis, poses diagnostic and management challenges, especially in patients with multiple comorbidities. A 58-year-old female with a history of poorly controlled diabetes mellitus, hypertension, hypothyroidism, coronary artery disease post-angioplasty, and stable chronic kidney disease presented with fever with chills, reduced appetite, dyspnea, and dysuria. A chest X-ray showed a blunted right costophrenic angle and ultrasonography revealed a moderate pleural effusion on the right side. Pleural fluid analysis confirmed tuberculous pleural effusion. Five days later, she developed slurred speech and a tingling sensation on the left side of her body. A computed tomography scan showed a left non-hemorrhagic lacunar infarct in the frontal lobe, which was confirmed by magnetic resonance imaging. Cerebrospinal fluid was negative for tuberculosis. She was started on antiplatelets for the infarct. Electroencephalography was normal. She had hypocalcemia and hyponatremia related to renal failure, which were also corrected. This case illustrates the challenges of managing tuberculous pleural effusion in patients with multiple comorbidities. Timely diagnosis and a comprehensive multidisciplinary approach are crucial for navigating the complexities of the case.

## Introduction

Tuberculosis (TB) remains a major public health concern in India, contributing to substantial morbidity and mortality. The *Mycobacterium tuberculosis* bacterium primarily targets the pulmonary system, resulting in pulmonary TB. However, the pathogen's capability to disseminate hematogenously allows it to infect virtually any organ system, leading to a variety of extrapulmonary TB manifestations. Among these, tuberculous pleural effusion is the second most prevalent form of TB, trailing only pulmonary TB in frequency [1,2]. Diagnosing and managing tuberculous pleural effusion is particularly challenging in patients with concurrent systemic diseases and multiple comorbidities, as these conditions can obscure clinical presentation and complicate treatment regimens. Effective management of tuberculous pleural effusion in such patients requires a multidisciplinary approach to address both the primary infection and the associated comorbidities, ensuring comprehensive care [3]. In this report, we present a case of an elderly female patient diagnosed with tuberculous pleural effusion, complicated by the presence of multiple comorbidities. This case underscores the necessity for heightened clinical vigilance and a tailored therapeutic strategy to manage TB in such a complex case.

## Case presentation

**Figure 1:**
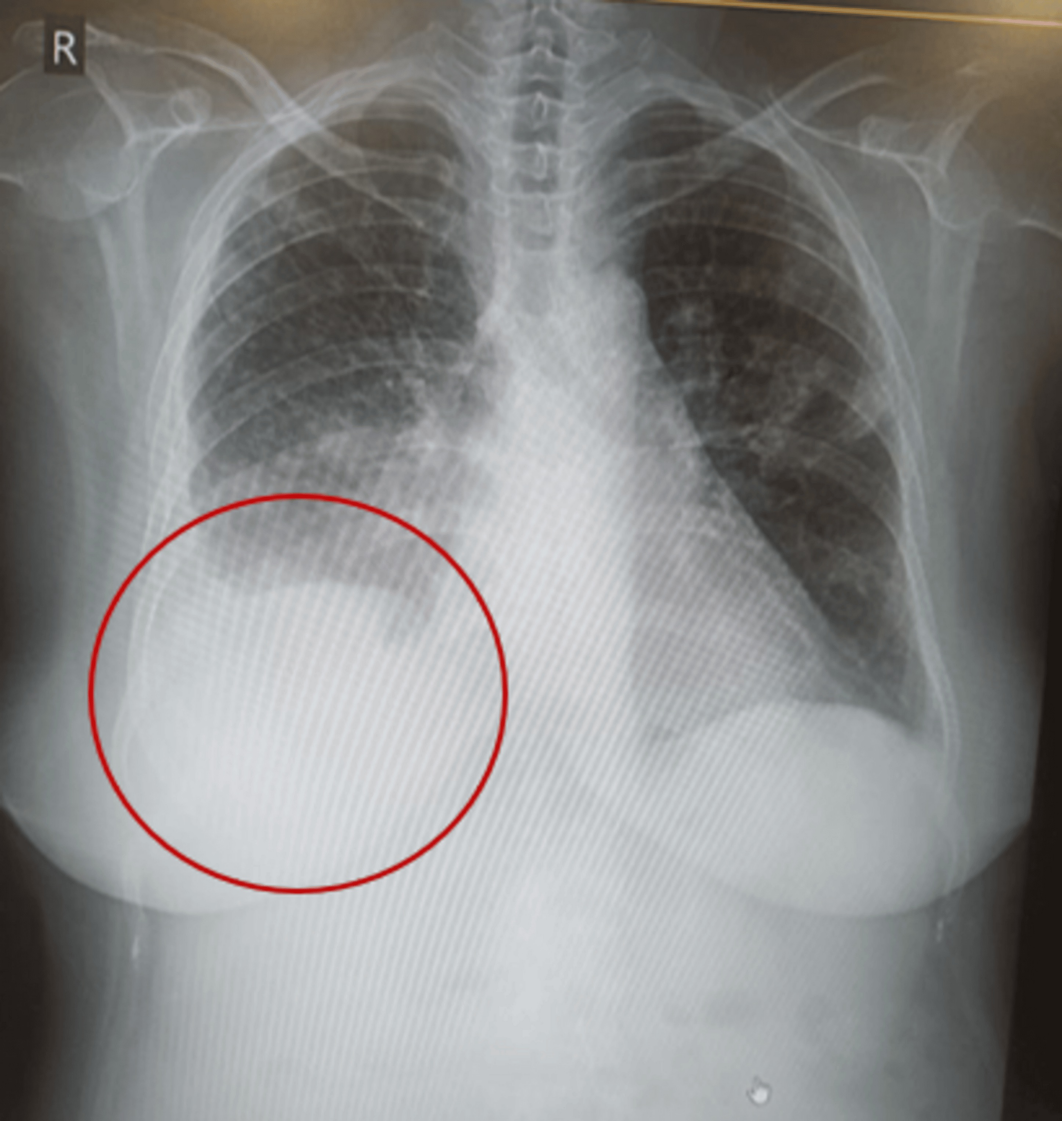
Chest X-ray showing blunted right costophrenic angle.

A 58-year-old female presented with intermittent fever with chills, reduced appetite, dyspnea, and dysuria for one week. She had a history of poorly controlled diabetes mellitus, hypertension, hypothyroidism, coronary artery disease with prior angioplasty, and stable chronic kidney disease with a glomerular filtration rate (GFR) of 28 ml/min/1.73 m^2^ body surface area. On presentation, she had hyperglycemia with a blood sugar level of 582 mg/dl and no ketoacidosis. On physical examination, she had normal blood pressure and heart rate, with no pallor, icterus, or cyanosis. She was conscious and oriented to time, place, and person with a Glasgow Coma Scale (GCS) score of E4 V5 M6. She had significantly reduced air entry on the right side of the chest, particularly in the lower right lung field, in the posterior and lateral regions. On evaluation, a chest X-ray showed a blunted right costophrenic (CP) angle, and ultrasonography (USG) revealed a moderate pleural effusion on the right side (Figure 1).

**Figure 2:**
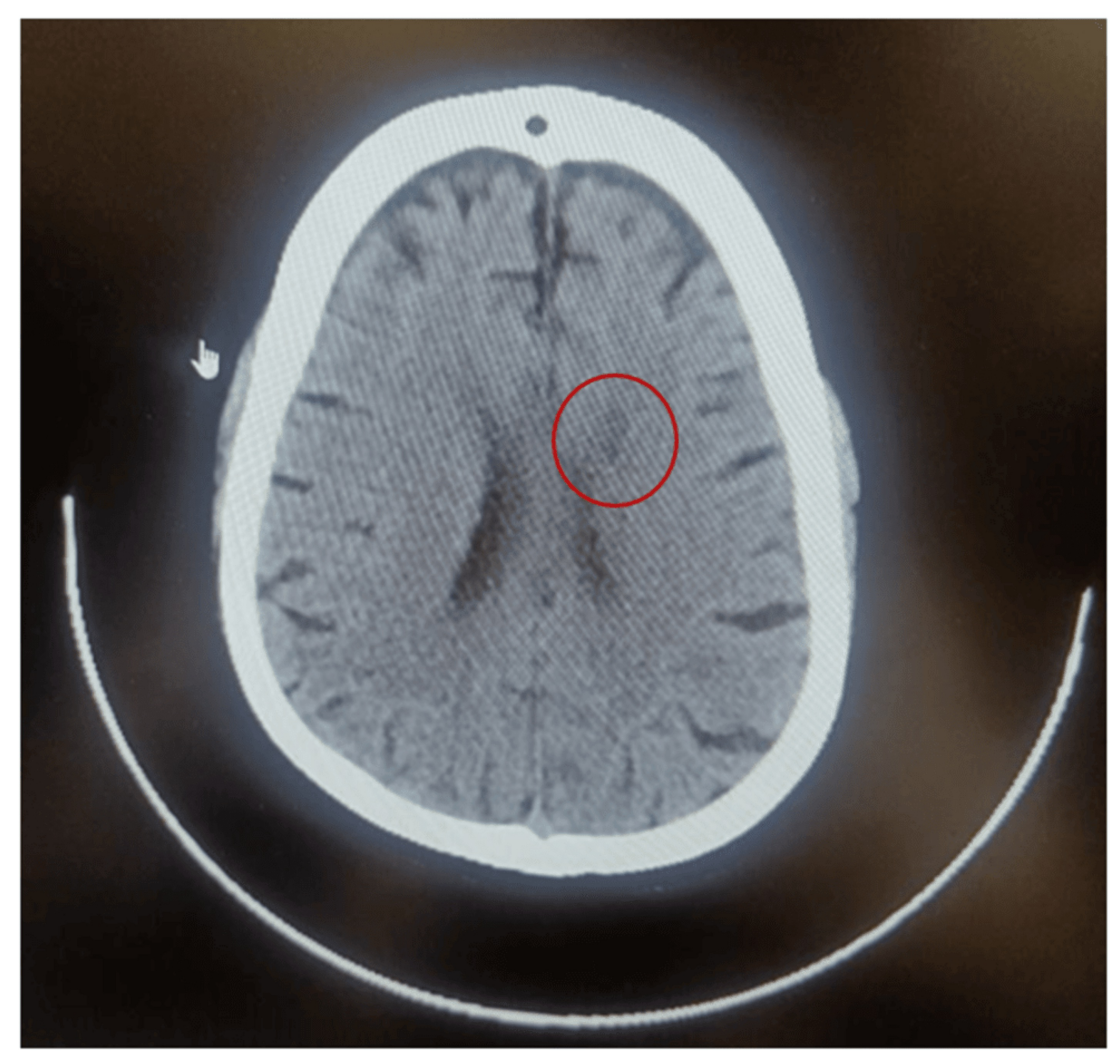
Computed tomography showing left non-hemorrhagic lacunar infarct in the frontal lobe.

**Figure 3:**
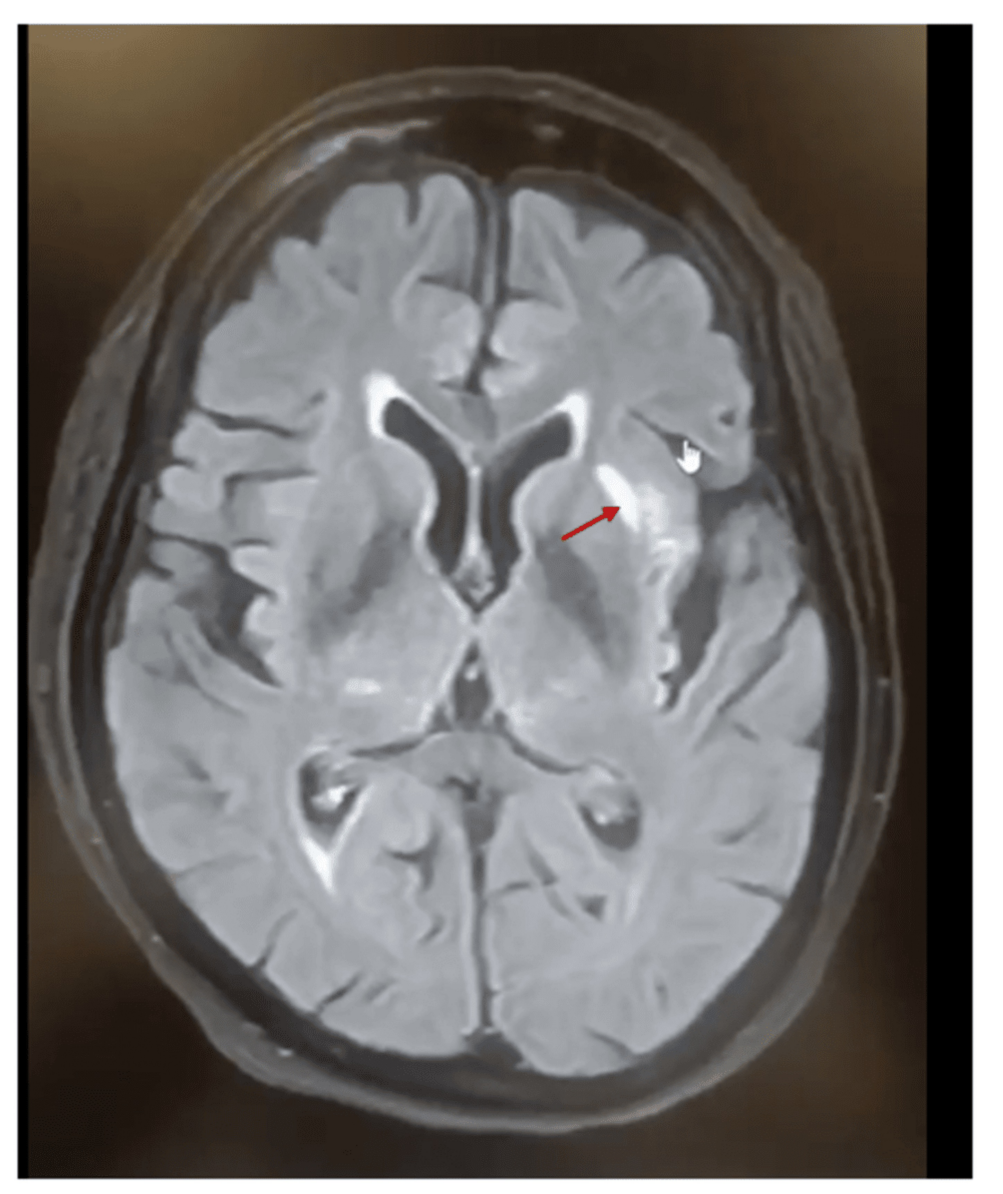
Magnetic resonance imaging confirming left non-hemorrhagic lacunar infarct in the frontal lobe.

A hemogram revealed anemia (hemoglobin: 9.9 g/dL) with normal white blood cell count and platelet counts. Anemia was attributed to chronic kidney disease. A two-dimensional (2D) echocardiography showed an ejection fraction of 50% (heart failure with preserved ejection fraction, class II). She was empirically started on intravenous meropenem and azithromycin injections. Blood sugar level was controlled (80-140 mg/dL) with insulin injections. Diagnostic and therapeutic pleural fluid tapping was performed on the right side of the chest. Pleural fluid was exudative with a leucocyte count of 1800/mm^3^, fluid protein of 3.9 g/dl, and fluid albumin of 1.7 g/dl. Differential diagnoses for an exudative pleural effusion with the mentioned leucocyte count could include infections such as bacterial pneumonia or TB, malignancies, pulmonary embolism, or autoimmune conditions like rheumatoid pleuritis or systemic lupus erythematosus. Fluid lactate dehydrogenase (LDH) was 921 U/L. Pleural fluid lighted criteria for exudative (pleural fluid protein to serum protein ratio > 0.5, pleural fluid LDH to serum LDH ratio > 0.6, and a pleural fluid LDH level exceeding two-thirds of the upper limit of the normal serum LDH level), with a pleural fluid protein to total protein ratio of 0.7 (>0.5), pleural fluid to serum LDH ratio of 4.0 (>0.7) (serum LDH value, 200 U/L), and a pleural LDH level >2/3 of the upper limit of the laboratory's reference range of serum LDH (120-240 U/L). The fluid had raised adenosine deaminase (ADA, 64 U/L), which is above the typical cutoff value of 40 U/L for diagnosing tuberculosis pleuritis. The fluid was positive for Xpert *Mycobacterium tuberculosis* (MTB). She was started on anti-tuberculosis treatment (5 mg/kg body weight (up to 300 mg) daily for up to nine months) for tuberculous pleural effusion. After five days of treatment, she developed slurred speech and a tingling sensation on the left side of her body. She had normal power in all four limbs, reduced tone in both ankle joints, normal tone in the rest of the joints, and a normal sensory examination. A computed tomography (CT) scan showed a left non-hemorrhagic lacunar infarct in the frontal lobe (Figure 2), which was confirmed by magnetic resonance imaging (MRI) (Figure 3).

Cerebrospinal fluid (CSF) was negative for TB (leucocytes = 2/mm^3^, negative Xpert MTB, ADA = 2.5 U/L, and normal protein and glucose levels). She was started on aspirin (initial dose: 160-325 mg, given as a single dose at the onset of symptoms or during the acute phase and for the infarct; maintenance dose: 75-100 mg daily, usually continued for one year). Electroencephalography (EEG) was normal. She had hypocalcemia and hyponatremia related to renal failure, which were also corrected. Hypocalcemia was managed with intravenous calcium supplementation and vitamin D to improve calcium levels. Hyponatremia was treated by fluid restriction or intravenous sodium replacement.

## Discussion

Prior to the COVID-19 infection, *Mycobacterium tuberculosis* was the most common infectious cause of mortality. It was only in the year 2021 that COVID-19 topped the list of infectious diseases causing deaths worldwide [1]. The prevalence of tuberculous pleural effusion is about 15-25% of all TB cases [1].

Tuberculous pleural effusion is more prevalent in immunosuppressed hosts such as diabetics, the elderly, and patients with chronic kidney disease. These patients often present with a more insidious presentation rather than an acute presentation [4]. Diabetes poses different challenges in managing tubercular pleural effusion. Tubercular pleural effusion is more common in diabetic patients than in non-diabetic individuals. Given that both diabetes and TB are highly prevalent diseases, their combination places a significant burden on the healthcare sector. In a study by Singh et al., the association between diabetes and tubercular pleural effusion was observed in 40% of patients [5].

Age is one of the least investigated risk factors for tuberculous effusion. Data on its incidence in the elderly are scarce, but elderly individuals represent a high-risk category with atypical presentation. Typical symptoms like pleuritic chest pain are less prevalent in the elderly. Radiographically, pleural thickening and calcification are more common presentations than fluid accumulation. In the elderly, a close differential diagnosis becomes malignancy, and delays in initiating anti-tubercular therapy have been observed [6]. In patients with chronic kidney disease, exudative pleural effusion or uremic pleural effusion is a close differential diagnosis for tubercular pleural effusion. Uremic pleural effusion is a more common cause of exudative pleural effusion in patients with chronic kidney disease than TB, especially in TB-endemic regions like India. As in diabetics and the elderly, CKD patients present a different diagnostic dilemma, such as a lower level of ADA, ADA being dialyzed to some extent, and poor yield in acid-fast bacilli (AFB) stain and culture [7]. Our patient had old age, diabetes, coronary artery disease, and chronic kidney disease as risk factors, placing her in a diagnostic dilemma category.

As far as complications are concerned, stroke is observed in 0.26 to 0.86% of patients with TB, but in almost all cases, it is associated with tuberculous meningitis. In patients with stroke and TB, it is very important to rule out TB meningitis, as patients with TB meningitis have poor outcomes compared to those with ischemic stroke. Our patient did not have TB meningitis, but it became necessary to rule out TB meningitis with radiology and CSF analysis as stroke due to TB vasculitis is located in different territories of the cortex, mainly lateral striate involvement, with damage to basal ganglia, cortex, or lobar, and internal capsule. Stroke due to TB vasculitis requires a prolonged duration of treatment with the addition of steroids to the treatment protocol and often shows a poor response to treatment. Therefore, an evaluation for TB meningitis is essential in patients with stroke and pulmonary or extrapulmonary TB.

## Conclusions

The presented case highlights the complexities involved in managing a patient with tubercular pleural effusion, concomitant infarct, and multisystem involvement. Tubercular pleural effusion, although a common disease, presents unique challenges, particularly in patients with comorbidities such as diabetes, chronic kidney disease, and advanced age. These factors not only complicate the diagnosis but also influence the treatment approach. Furthermore, the case underscores the significance of a multidisciplinary approach in managing such complex patients. Collaboration among pulmonologists, neurologists, nephrologists, and other specialists is crucial to ensure comprehensive care and optimal outcomes.
